# Supramolecular and Liquid Crystalline Contributions to the Assembly of Myofibril

**DOI:** 10.3390/molecules25040862

**Published:** 2020-02-15

**Authors:** Alberto Ciferri, Alvin L. Crumbliss

**Affiliations:** Chemistry Department, Duke University, Durham, NC 27708, USA; alvin.crumbliss@duke.edu

**Keywords:** myofibrillogenesis, chemical recognition, shape recognition, supramolecular polymerization, liquid crystallinity

## Abstract

We compare steps observed during the fibrillogenesis of myofibrils with the sequence of steps predictable by a recent analysis of the structurization and functioning of striated muscles. The predicted assembly steps are based solely on fundamental equilibrium processes, particularly supramolecular interactions and liquid crystalline alignment of the rigid thick and thin filaments hosted within the sarcomer. Satisfactory agreement is obtained between several of the observed and the predicted fibrillogenesis steps. In several cases, however, the actual steps appear to be more complex than expected, evidencing the occurrence of transport and kinetic pathways that may assist the attainment of the equilibrium structure. The memory of the order of a precursor mesophase is imprinted during the remodeling of the surfaces at which the two sets of filaments are anchored. The relevance of the present analysis to the functioning of the myofibril is considered.

## 1. Introduction

The in vivo stepwise assembly of a complex biochemical structure starts with the biosynthesis of various proteins having specific sequences that ought to be evolutionarily coded for the manifestation of the final, functional properties. The ensuing step is the organization of the components into assemblies consistent with the secondary and tertiary structure of the components. The organization of these assemblies into superstructures is a complex step that may involve a large number of proteins, some of which may have regulatory functions and are transiently present.

**Figure 1:**
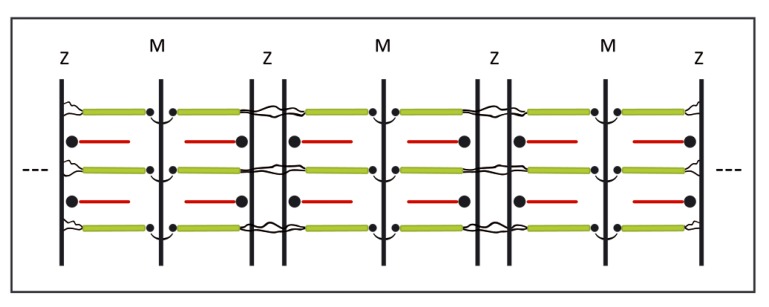
Schematic representation of three sarcomeric repeating units along the polymeric myofibril. The contracted state of the muscle is evidenced by the interpenetration of the thick (green) and the thin (red) filaments. The thin filaments are based on rigid actin molecules bound to tropomyosin and troponin and anchored to the Z-zone by the cross-linking protein α-actinin. The thick filaments are based on rigid myosin molecules bound to up to six semi-flexible titin molecules (dark) bound to the M line and to the Z zones. Each sarcomer includes two half-sarcomers specularly connected at the M line. Titin is the only component that spans the entire length of each sarcomer and of the myofibril. Neighboring sarcomers polymerize by bridges of α-actinin that links two titin NH_2_ terminals that emanate from each thick filament. An elongated membrane envelops the myofibril.

The biosynthesis and the biochemical signals that trigger the accretion of the constituent proteins have been investigated by sequence and other sophisticated biochemical studies. However, the fundamental nature of the steps between the biosynthesis and the appearance of the final structures are frequently not described. For instance, the self-assembling mechanism of the basic repeating unit of the myofibril (the sarcomer, see Figure 1), remained obscure during over a half century of investigations on striated muscles. A complex model for muscle contraction based on ATP-dependent interactions between actin and the motor myosin filament remained unchallenged, in spite of relevant advances in the area of fundamental self-assembling mechanisms [1,2,3,4].

In recent communications, we analyzed the structure and the contraction mechanism of the myofibril. We highlighted the basic physico-chemical principles likely involved in both the assembling and the functioning of the sarcomere [3,4]. In particular, the interdigitated distribution of thick and thin filaments in the contracted state was suggested to reflect a memory of a liquid crystalline order of mixtures of the two unrestricted components in the presence of an excess of Ca^2+^ ions. The swing away of the two set of filaments during relaxation was instead confirmed to reflect the electrostatic repulsion of the two polyanionic filaments unscreened by mobile counter-ions. The linear organization of the sarcomeric components along the fiber axis was attributed to a series of chemical cross-linkages engineered by Nature to optimize the mechanical function of the muscle. The driving step in the assembly of the myofibril was postulated to be the supramolecular polymerization of the sarcomers [4]. In the present communication, we compare the assembly steps predicted by our model with steps documented by direct analyses of the myofibrillogenesis process and the structure of mature myofibris [5,6,7,8,9].

## 2. Roles of Chemical and Shape Recognition

The stability of any structure (molecular or macroscopic) requires attractive forces that maximize the adhesion between the building blocks (chemical recognition), and geometrical compatibility of these building blocks, which minimize their excluded volume (shape recognition) [10,11]. The following short summary underlines the fundamental theoretical concepts of chemical and shape recognition processes. The detailed concepts allowed the recent prediction of the equilibrium structure of the myofibril [3,4].

### 2.1. Chemical Recognition

(A) Mono-functional units produce closed complexes. Bifunctional units produce supramolecular polymers. Depending upon the number, strength and orientation of the supramolecular bonds, linear chains or helical polymers with large polymerization degree may be produced [10,11,12]. Subsequent transformation of supramolecular into covalent bonds may occur.

(B) Ionic interactions. In addition to the conventional electrostatic interaction between fixed and mobile charges, “ion condensation” effects promote an attractive parallelization of rigid chains sharing bivalent counter-ions [13,14,15].

### 2.2. Shape Recognition

(C) Liquid crystals. Excluded volume effects promote the mutual alignment of rod-like neutral polymers at a critical volume fraction of the solute (v^c^) for covalent [16,17] and supramolecular polymers [18].

(D) Flexible segments are excluded from the LC phase of rigid polymers [17].

### 2.3. Mixtures

(E) Compatibility is exhibited by liquid crystalline mixtures of rigid neutral polymers [17].

(F) Incompatibility exhibited by liquid crystalline mixtures of rigid polyelectrolyte carring a similar charge [19,20,21,22].

(G) Surface anchoring of a liquid crystal results in a further restriction to the lateral displacement of the first layer of anchored molecules [23].

## 3. Comparison Between Actual and Postulated Assembling Steps

### 3.1. Formation of the Thin and Thick Filaments

The first step in the fibrillogenesis process is the availability of the basic monomers for the production of the main polymeric components: actin, myosin and titin. The assembly of actin occurs by supramolecular polymerization, as first suggested and experimentally demonstrated by Oosawa (point A above). However, actin and myosin entering the thin and the thick filaments are actually bound to other polymers. The thin filament is a complex of actin that includes minor amounts of tropomyosin and troponin. The thick filament is instead a complex of several myosin rigid dimers terminated by globular heads (sites for ATP-phase having a fundamental role in the Huxley’s model for muscle contraction [1,2]), and three to six titin molecules. The latter polymer has a conformation characterized by unstructured sequences and structured domains [1]. The winding of titin around the thick segment was well characterized and the specific sequences involved in these interactions were often identified [7].

In terms of our model, the formation of the myosin-titin complex represents a strategic assembling step that must occur before the insurgency of the liquid crystalline order. In fact, titin spans the entire sarcomer length between its Z zones: its semi-flexible conformation would not be admitted into a liquid crystalline phase (point D above). If the proper complementary sequences had been imprinted during the biosynthesis, the formation of the titin-myosin complex would simply occur in by chemical recognition of the binding sites in the isotropic solution.

Some of above expectations were verified by myofibrillogenesis studies on developing skeletal muscle cells [5]. Wang and al. reported that α-actinin was present at all development stages and able to bind to the multiplicity of proteins (up to 25) that appeared during the mature stage [6,7]. Titin appeared in the early stages of the myofibril assembly, supporting our expectation. However, myofibrillogenesis studies, such as those reported by Pizon et al. [8], suggest that a compound labeled MURF2, associated to microtubules, directs the myosin-titin association. Remarkably, the MURF2 complex disappears from the sarcomer following the alignment of the tick filament. Our model does not readily explain the role of MURF2. Nevertheless, the recovery of the postulated alignment of tick filaments in the mature myofibril suggests a possible slowdown on the formation of the equilibrium structure by the kinetic pathways of the assembly process.

### 3.2. Development of Liquid Crystallinity

Consistent with our model and with EM observations in embryonic material, the protein components and the nascent thick and thin filaments were found to be initially dispersed in an isotropic solution within the cytoplasm [9]. An increase of their concentration should eventually trigger the formation of a liquid crystalline phase (point C above). If an excess of screening Ca^2+^ ions is present, a single liquid crystalline phase would be expected to include both thick and thin filaments (point E above). Ion condensation effects would contribute to the stabilization of the mixture (point B above). Under the optical microscope, the cytoplasm should appear to be depolarizing, reflecting the occurrence of ordered domains with a random director orientation [22]. The elastic sections of titin ought to be excluded from the mesophase, as theoretically predicted (point D above).

We have not been able to find studies establishing the actual formation of the predicted liquid crystallinity in embryonic liquids. The only support we can offer is associated with the evaluation of the axial ratios of the thick and thin filaments that are significantly larger than the theoretical value at which liquid crystalline phases are stabilized [3]. A strong indirect support for the occurrence of a precursor liquid crystalline phase is the observation of EM micrographs for mature myofibrils (see Figure 1). Ishiwata and al. have shown that the latter structure can be described as a smectic A-type structure along the fiber axis (with the sarcomers aligned along the axis), whereas a hexagonal organization in the filaments prevails within a cross-section of the overlap region [24]. It would be difficult to justify the formation of such a smectic organization without admitting the formation of a precursor nematic phase [11,17].

### 3.3. Assembly of the Sarcomer

The random director orientation of a multi-domain liquid crystalline solution would be an obstacle to the development of a sarcomer that displays contractile behavior along a single fiber axis [3]. The strategy used by Nature to secure one-dimensional contraction is the anchoring of the liquid crystalline components of each sarcomer to surfaces at the Z zones and the M line. Anchoring to a surface is frequently used for practical applications of liquid crystals (point G above).

Particularly relevant are myofibrillogenesis studies revealing the sequence of association of thin filament to the Z zone, and of the thick filament to the M line. The studies by Wang and coworkers revealed that Z-bodies, beginning to form during the pre-myofibrillar stage, were remodeled during the following nascent and mature stages, and accumulated along the sarcometer membrane forming complexes with actin and α-actinin. Proteins such as myomesin and M-protein were instead found to anchor titin and the thick filaments to the M-line, possibly bridging the titin COOH terminals of the two half sarcomers [6]. Remarkably, the thick filaments were found to assume their final orientation before the thin ones [8].

**Figure 2:**
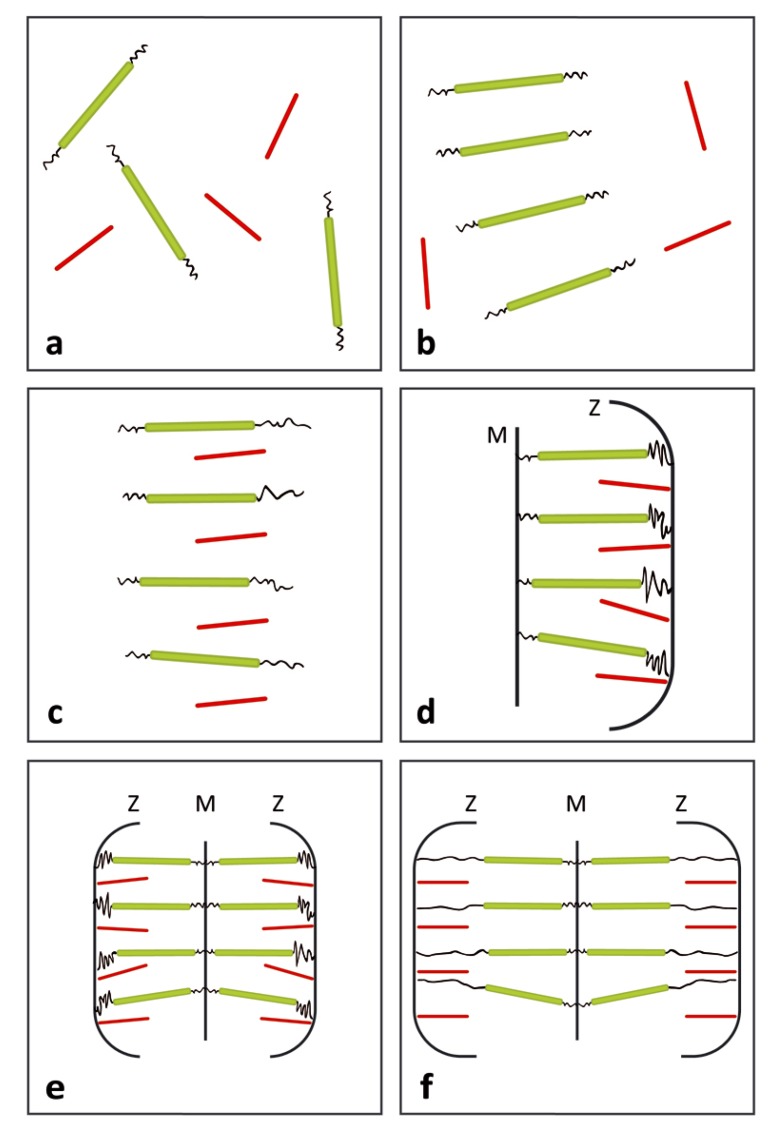
Suggested sequence of assembly steps during the formation of individual sarcomeres. Color code as in Figure 1. (**a**) The first stage is regarded as a cell hosting a single-phase isotropic solution with volume fractions of the thick and thin filaments below their corresponding critical values v^c^. (**b**) Upon increasing filament production, the critical value of the thick filament is attained and the cell hosts a biphasic dispersion including the liquid crystalline phase of the latter filament separated from the isotropic phase of the thin one. (**c**) Upon further increase of filament production, the critical concentration will be attained also by the thin one. Under an excess of Ca^++^ ions, both filaments are electrostatic ally screened and able to form a single mesophase. (**d**) Anchoring of the two types of filaments to opposite sides of the cell defines the remodeling Z-zone and M line in which the exact spacing between the two filaments is eventually imprinted. (**e**) Specular coupling of two half-sarcomers at the M line produces a full sarcomer. (**f**) Pumping Ca^++^ ions out of the sarcomer produces reversible elongation of the cell manifested by separation of the thick and thin filaments and elongation of elastic titin segments.

The above results are in line with our model expectations and with scattered fibrillogenesis data, and offer plausible support the following sequence of assembling steps. The thick filaments are the first to assume liquid crystalline order. When the concentration of the thin filaments reaches the value for phase separation, both filaments interdigitate into a single liquid crystalline phase provided an excess of Ca^++^ ions is present. The complete formation of the Z zone and the M line set the final spacing of the two types of filaments. Alternatively stated, the memory of the interdigitated topology is imprinted during the final remodeling of the anchoring surfaces. The bonds at the M line allow the specular assembly of two half-sarcomers and the occurrence of a common axis for the contraction-relaxation cycles of the whole sarcomer. Removal of Ca^++^ ions will trigger demixing into two liquid crystalline phases (relaxed state, point F above). The above sequence is illustrated in Figure 2 that offers a schematic representation of the main development stages during the assembly of the sarcomer.

### 3.4. Assembly of the Myofibril

The assembling step of the myofibril ought to assure the continuity of a linear structure that allows a coordinated performance of the contraction-relaxation cycles. To this end, individual sarcomers need be oriented and connected by strong linkages along the fiber axis. The linear assembling mechanism of the sarcomers remained unexplained due the difficulty in evidencing a bond connecting the sarcomers. Recently, we postulated a polymerization mechanism based on a direct bridge between α-actinin and the NH_2_ titin terminals of adjacent sarcomers [3]. An analysis by Liversage et al. offered evidence for the postulated bond [25]. They compared the actual symmetry parameters of the thick and thin filaments with the corresponding ratio of the number of these filaments. They concluded that only four terminals of the six titin molecules emerging from each thick filament could react with actin residues in the Z-zone within a given sarcomer. The remaining two could enter the adjacent sarcomers (see Figure 1) [25]. The energetic of such a bond was adequate to justify the actual average length of the myofibril in terms of the theory of supramolecular polymerization [3,12].

The final stage of the assembly of individual myofibrils is the fusion of the individual sarcomer membranes into an elongated membrane having coordinated channels for the diffusion in and out of Ca^++^ ions. The formation of the latter membrane represents an additional stabilization due to supramolecular polymerization. In fact, it has been shown that linear growth of anisotropic micelles is simultaneous with their parallelization into ordered mesophhases [1,18].

The myofibrils further assemble in the parallel array of myofibers, thus leading to the geometrically well-defined shape of the skeletal muscle. It is to be remarked that in the case of the cardiac muscle a network-type of myofibrillar organization is instead required to adapt the contraction-relaxation mechanism of the sarcomers to the complex shape and functions of the hearth [26].

## 4. Relevance to the Dynamic Function of the Myofiber

The original “sliding filament” treatment introduced by Hanson assumed that Ca^++^ ions pumped from the sarcomplasmic reticulum (SR) entered the sarcomer membrane and established a direct attractive interaction between the thin and the tick filaments [1,2]. The postulated “salt bridges” drove the thin filaments toward the center of the sarcomer. Relaxation occurred when the Ca^++^ ions were pushed back to the SR. The ion condensation effect we introduced appears to be a modern expression of the poorly defined salt bridges postulated by Hanson.

The “swinging cross-bridge hypothesis” was later introduced by Huxley. He revised the contraction step and postulated the formation of direct supramolecular bonds between the myosin heads and binding sites on the thin filament [1,2]. The latter bonds involved α-actinin and were formed and broken at consecutive locations due to the combined interaction of Ca^++^ and ATP.

The swinging cross-bridge model introduced by Huxley is a perplexing one. It was proposed at a time when the liquid crystalline behavior of rigid rods, based on fundamental matter properties, was unknown. Today, the idea that actin makes discrete steps in myosin is a useful concept in cell motility [27]. Indeed, motor properties have been evidenced for a myosin II model [28]. Nevertheless, the compatibility of the screened filaments within a single mesophase, coupled to ion-condensation effects, should definitively aid the contraction step. It is to be noted that the interaction between ATP and Ca^++^ is essential also to the exchange of Ca^++^ ions between the sarcomer and the SR. Working with striated muscles, Stokes evidenced that the re-*uptake* of Ca^++^ ions by the SR requires a coupling of the energy of the ATP hydrolysis to calcium transport [29]. Therefore, the often-cited evidence that in the absence of ATP the muscles do not relax (rigor mortis) [9] is not inconsistent with our model.

Refined quantitative analyses are needed to assess the extent of all contributions to the dynamic functioning of the myofiber. The process may also be described in the context of the Complexity paradigm [4].
